# Supplementary material for: Learning to express reward prediction error-like dopaminergic activity requires plastic representations of time
Source: Nat Commun. 2024 Jul 12;15:5856. doi: 10.1038/s41467-024-50205-3 (PMC11245539; doi:10.1038/s41467-024-50205-3)
Supplement: Supplementary file 1 — Supplementary Information [file 41467_2024_50205_MOESM1_ESM.pdf]

Supplemental Materials

Learning to Express Reward Prediction Error-like Dopaminergic Activity Requires Plastic Representations of Time

Abbreviated title: RPE via Plastic Representations of Time

Ian Cone[1,2,3], Claudia Clopath[1], Harel Z. Shouval[2,4]
[1]Department of Bioengineering, Imperial College London, London, United Kingdom;
[2]Department of Neurobiology and Anatomy, University of Texas Medical School at Houston, Houston, TX,USA;[3]Applied Physics Program, Rice University, Houston, TX, USA;
[4]Department of Electrical and Computer Engineering, Rice University, Houston, TX, USA

Supplemental Figures

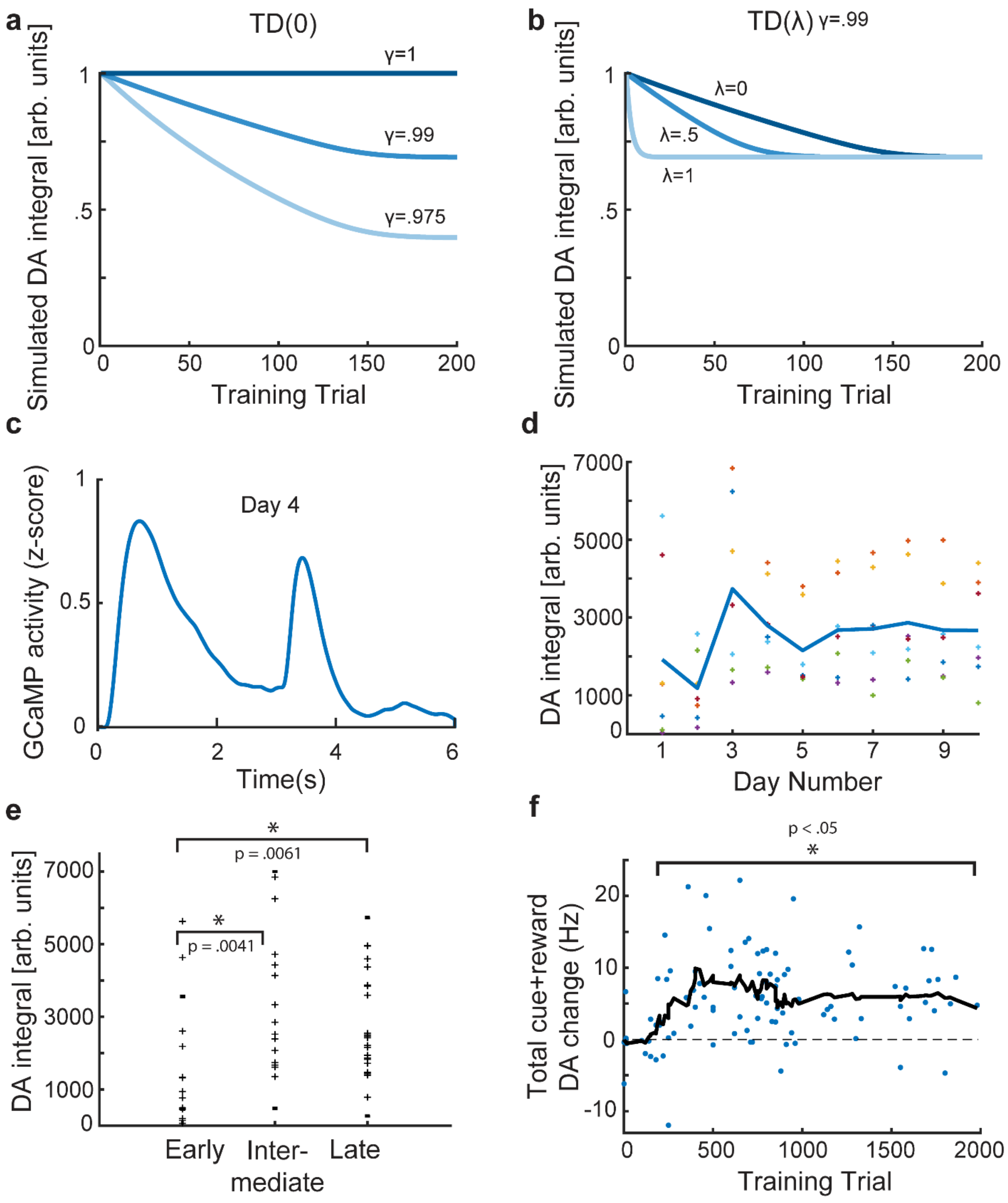

Supplemental Figure 1 – Experimentally observed dynamics of the DA integral vs those predicted by FLEX and TD.

a) Integrated dopamine (DA) over learning for temporal difference learning (TD(0)), given different values of γ. **b)** Integrated DA over learning for TD(λ), given different values of λ, with γ fixed at .99. **c)** Time course of Z scored GCaMP signal per one animal and one day from the Amo et al data (2022)[27] (blue curve). The area under the curve is the definition of the integrated signal. **d)** Re-analysed data from Amo et al (2022)[27]. Here we plot the

time evolution of the integral of DA response over training days. The different color symbols are the average DA integral (averaged over trials) per each training day, per each animal. Different animals are color coded. The blue line is the mean over animals. **e)** Average integrals for early (days 1-2), intermediate (days 3-4) and late (days 8-10) of training. The integral in the early period is significantly lower than in the intermediate and the late periods (Wilcoxon rank sum test: p=0.004 between early and intermediate and p=0.006 between late and intermediate), but intermediate is not significantly higher than late. **f)** Horizontal axis is the training trial, and the vertical axis is the mean activity modulation of DA neuron activity integrated over both the cue and reward periods (relative to baseline). Each blue dot represents a recording period for an individual neuron from either ventral tegmental arear (VTA) or substantia nigra compacta (SNc) (n = 96). The black line is a running average over 10 trials. Bracket with star indicates blocks of 10 individual cell recording periods (dots) which show a significantly different modulated DA response (integrated over both the cue and reward periods) than that of the first 10 recording periods/cells (Significance with a two-sided Wilcoxon rank sum test, p<0.05).

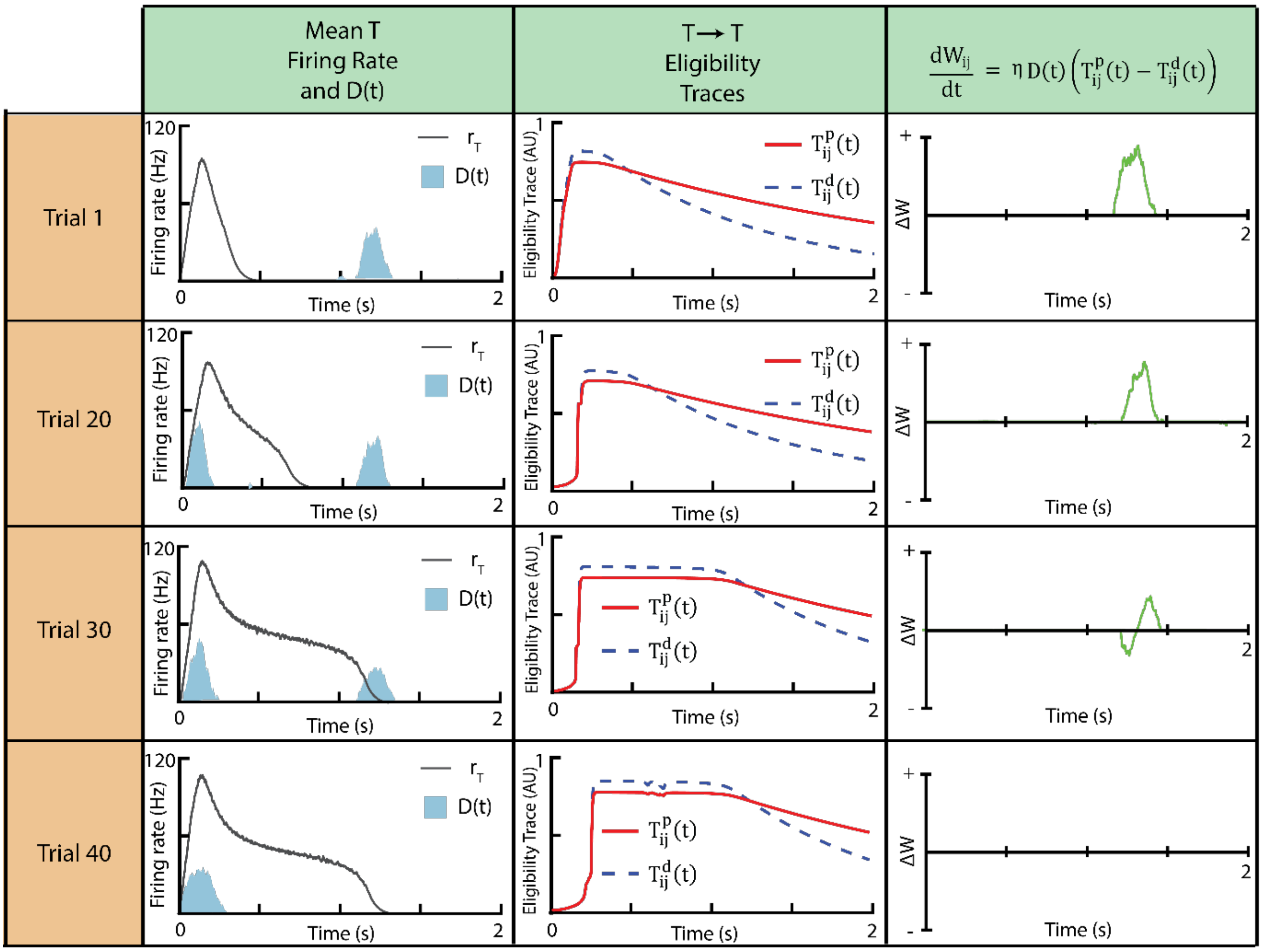

Supplemental Figure 2 – Two-Trace Learning Flexibly Encodes Cue-Reward Delay in Recurrent Connections of Timer Neurons Demonstration of the dynamics of two-trace learning for recurrent connections. Rows consist of different trials. Left column, mean firing rate of Timer neurons (black) and dopamine reinforcement D(t) (light blue) for a given trial. Middle column, long-term potentiation LTP (red) and long-term depression LTD (blue) associated eligibility traces triggered by the Hebbian overlap $\frac{r_i \cdot r_j}{1+\alpha D(t)}$. Right column, $\frac{dW}{dt}$, calculated at a given time as the difference between the two traces $(T^p_{ij} - T^d_{ij})$ multiplied by the dopamine reinforcement, D(t). For all trials, the increase in recurrent weights is mediated by dopamine release at t_{US}. Since dopamine acts to suppress trace generation in prefrontal cortex (PFC), the conditioned stimulus evoked (CS-evoked) dopamine response (trial 20) has little effect on the recurrent learning. The weights increase until ΔW is zero (trial 30) and remain at their fixed point after unconditioned stimulus evoked (US-evoked) dopamine has been suppressed (trial 40).

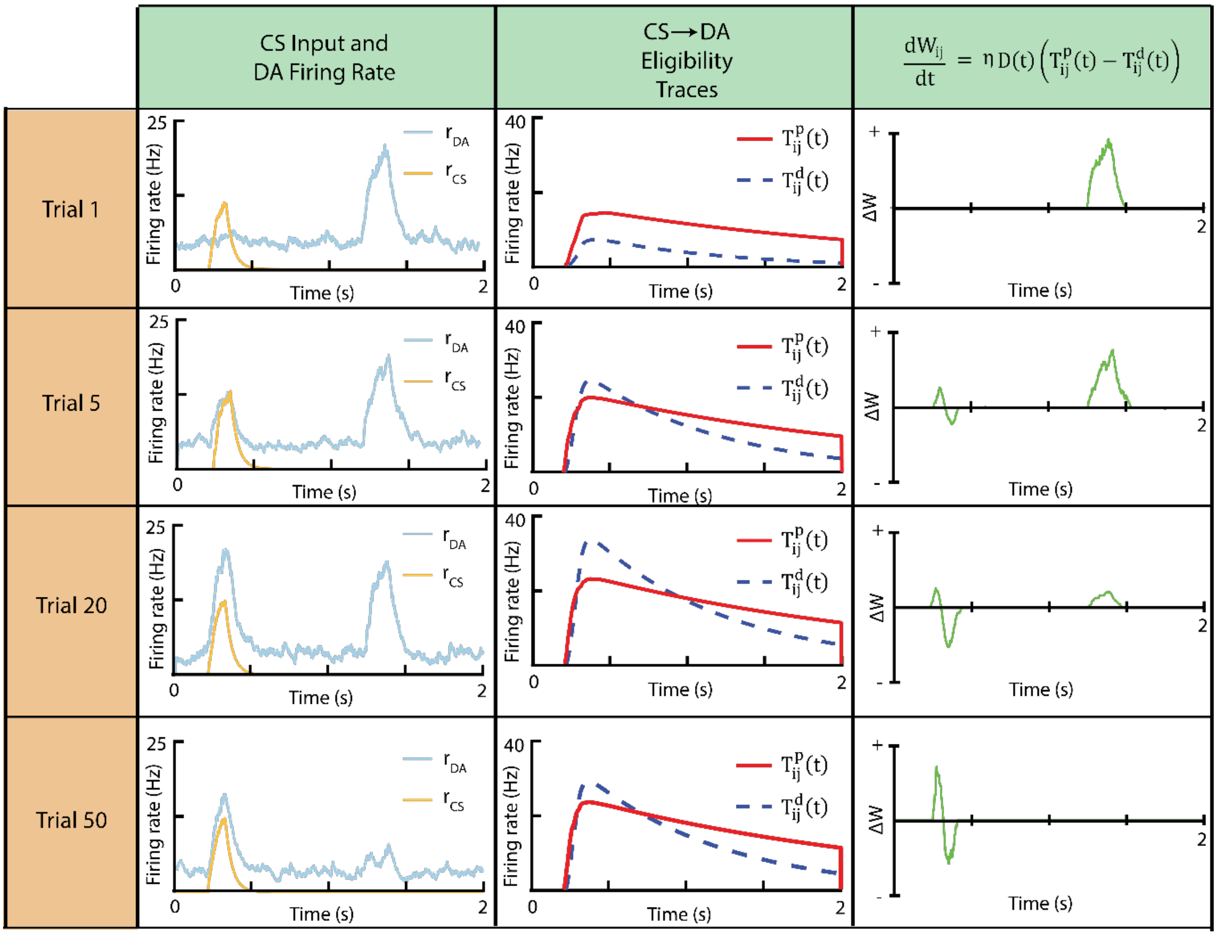

Supplemental Figure 3 – Two-Trace Learning Encodes Reward Predictive Cues via Feed-Forward Connections to DA Neurons

Demonstration of the dynamics of two-trace learning for feed-forward connections. Rows consist of different trials. Left column, input from external conditioned stimulus (orange) and mean firing rate of dopamine neurons (light blue) for a given trial. Middle column, long-term potentiation (LTP, red) and long-term depression (LTD, blue) associated eligibility traces triggered by the Hebbian overlap $r_{CS} * r_{DA}$. Right column, $\frac{dW}{dt}$, calculated at a given time as the difference between the two traces ($T_{ij}^p - T_{ij}^d$) multiplied by the dopamine reinforcement, D(t). Initially (trial 1), increase in feed forward weights is mediated by dopamine release at t_{US}. However, as a conditioned stimulus(CS) evoked dopamine response begins to develop (trial 5), the weights are bounded at a fixed point, constrained by the relative positive (from the unconditioned stimulus (US) and negative (from the CS) contributions (trial 20). After the expected reward at t_{US} has been depressed, the DA neuron firing at t_{CS} decreases slightly until $\frac{dW}{dt}$ reaches a fixed point maintained by the CS dopamine alone.

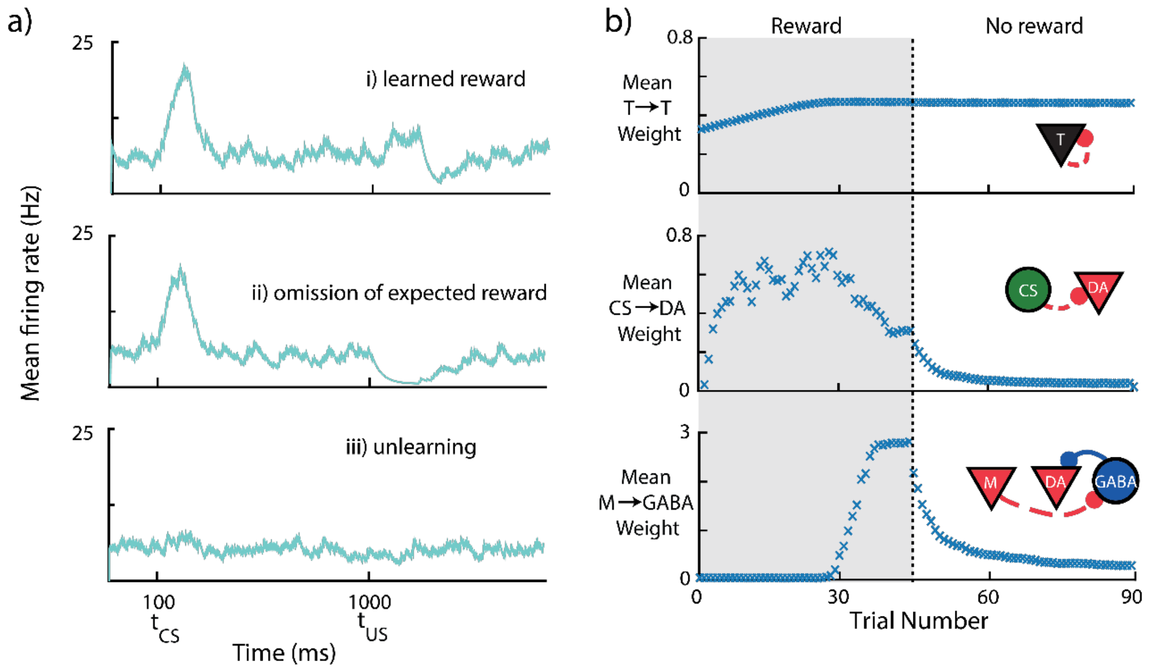

Supplemental Figure 4 – Consistent Unpairing Leads to Negative RPEs and Unlearning
a) Mean firing rate over all ventral tegmental area (VTA) dopamine (DA) neurons for a given trial after learning reward (i), upon omission of the learned reward (ii), and finally, following unlearning (iii). The omission of the expected reward produces a characteristic dopamine "dip", which in turn acts as a negative reward prediction error (RPE) and facilitates unlearning of the cue-reward association. **b)** Mean Timer→Timer (top), CS→DA (middle), and M→GABA (bottom) synaptic weights over the course of pairing and unpairing. The cue is presented for all trials, while the reward is only presented for the first 45 trials (shaded grey). For trials ~30-45, M→GABA weights increase, suppressing the amount of DA firing at the time of the reward. In response, the fixed point of CS→DA weights (which is determined, in part, by the dynamics of the dopamine reinforcement D(t)) decreases before stabilizing in trials ~40-45. Following omission, both CS→DA and M→GABA weights decrease to zero, but T→T weights are maintained, since the fixed point for these weights (see Supplemental Figure 2) is agnostic to changes in to D(t). Definitions: dopamine releasing neurons (DA), timers (T), messengers (M), conditioned stimulus (CS), inhibitory neurons (GABA).

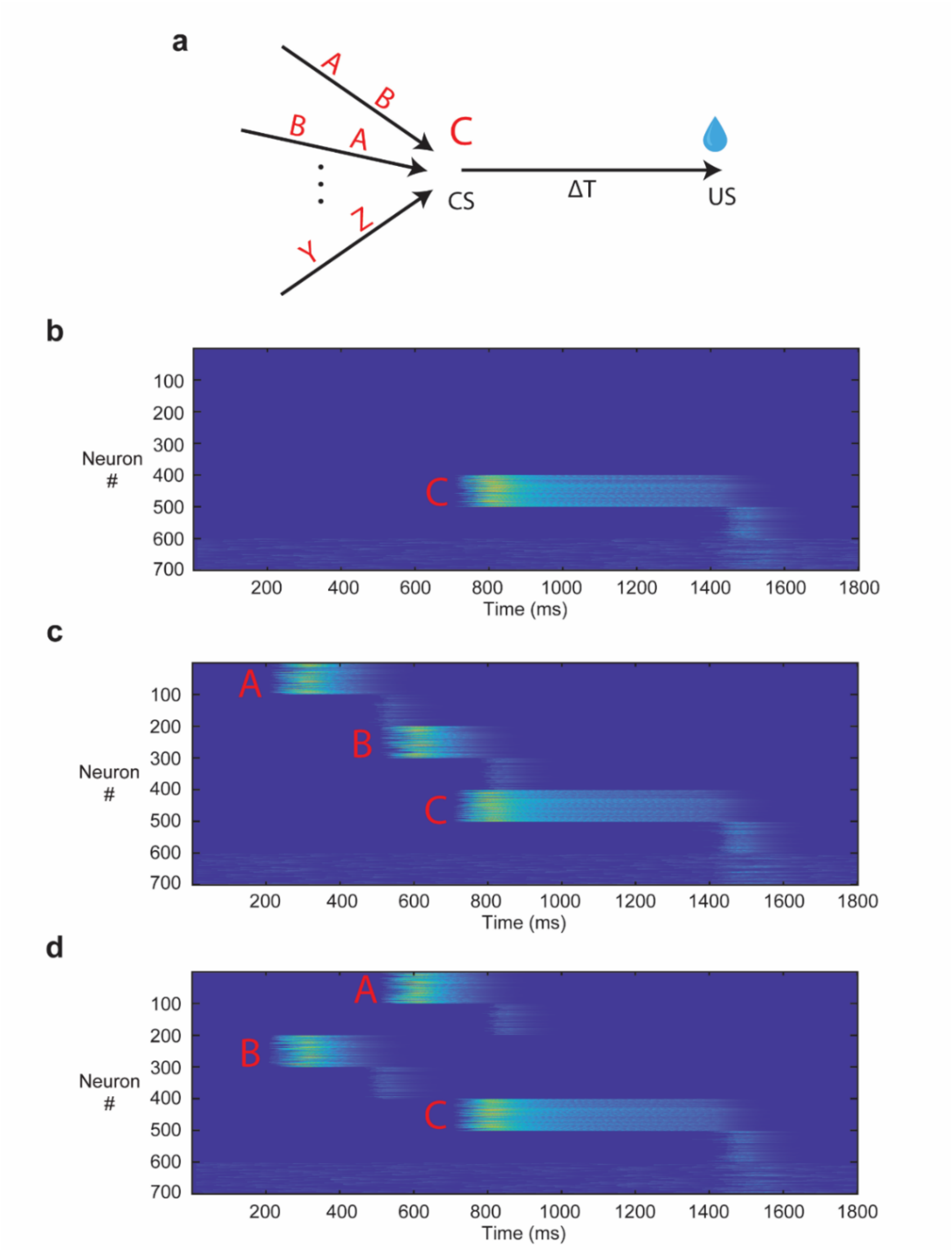

Supplemental Figure 5 – Distractor Cues in FLEX model do not disrupt learned timing
a) Schematic of the task protocol. Every presentation of C is followed by a reward at a fixed delay of 750ms. However, any combination or sequence of irrelevant stimuli may precede the conditioned stimulus C. In Figure 2, we showed that in this task, a fixed RNN has its timing information relative to C disrupted by these “distractor” cues. **b-d)** Firing rates of neurons in the FLEX model after training on the task. **b)** The presentation of cue C leads to the activation of C-related Timers (neurons 401-500) and C-related messengers (neurons 501-600), accurately reporting the 750ms delay. **c)** Presentation of cue C is preceded by cues A (at time 200ms) and B (at time 450ms). The learned timing between cue C and the reward is unaffected. Both cues A and B are randomly interleaved during training but have no pairing with reward. **d)** The same setup as in c), but with the order of the distractor cues reversed.

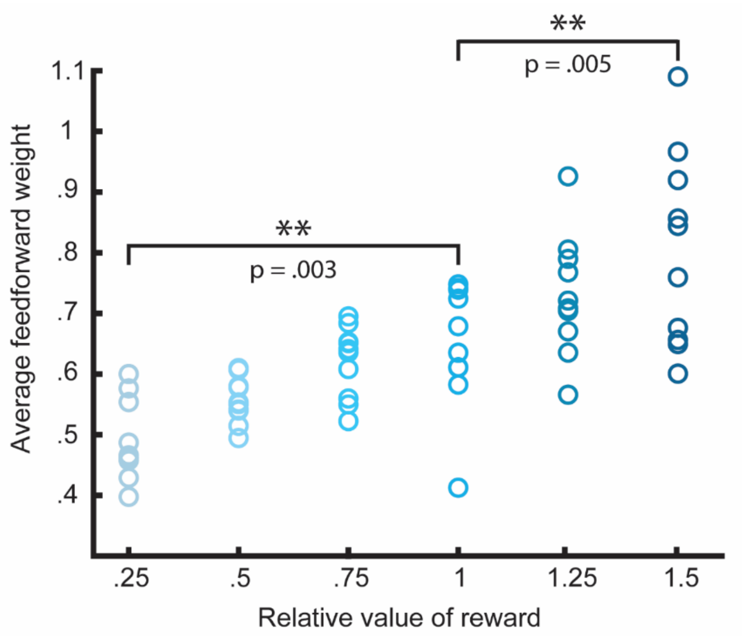

Supplemental Figure 6 – Modified FLEX model learns to encode reward magnitude in cue-evoked DA response

The average feedforward (CS->VTA DA) weights in the network as a function of the relative value of reward, in an alternate version of our FLEX model. In this alternate version, we assume that other neuromodulators are released alongside dopamine upon reward, such that when rewards are predicted, dopamine release itself is suppressed while the release of these other neuromodulators is not. This allows for the network to encode the reward magnitude in its cue-evoked DA responses, even after training. For each reward magnitude, we ran 10 different simulations from random seeds. The means (over simulations) of the feed-forward weights corresponding to the smallest (.25) and largest reward (1.5) magnitudes were found to be statistically significantly different from the mean (over simulations) of the feed-forward weight in the control group (reward value = 1), using a one -way ANOVA test. Definitions: conditioned stimulus (CS), dopaminergic neurons in ventral tegmental area (VTA DA).

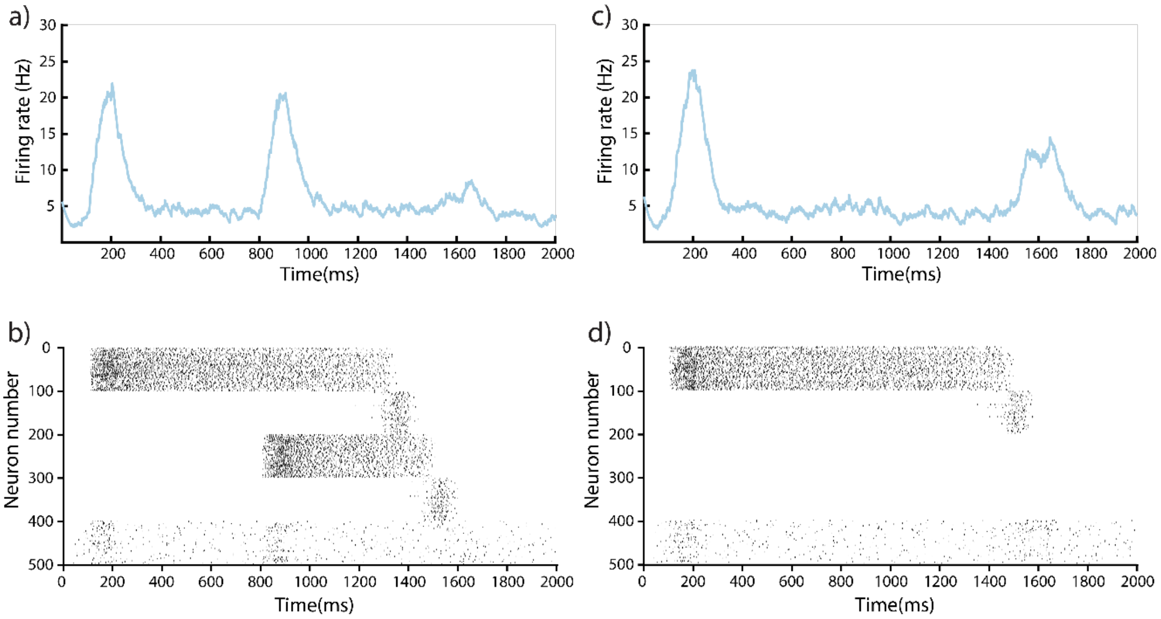

Supplemental Figure 7 – Both Cues Contribute to Reward Prediction Following Initial Training

a) Mean firing rate of all VTA DA neurons for presentation of CS1+CS2+US following sequential conditioning. **b)** Spike raster of Timer neurons (1-100, 201-300), Messenger neurons (101-200, 301-400), and VTA DA neurons (401-500) for the same trial. **c)** Mean firing rate of all VTA DA neurons for presentation of CS1+US, omitting CS2 following simultaneous conditioning. Notably, CS1 only partially predicts (and in turn partially inhibits) the dopamine response at t_{US}. d**)** Spike raster of Timer neurons (1-100, 201-300), Messenger neurons (101-200, 301-400), and VTA DA neurons (401-500) for the same trial. Definitions: first conditioned stimulus (CS1), second conditioned stimulus (CS2), dopaminergic neurons in ventral tegmental area (VTA DA).

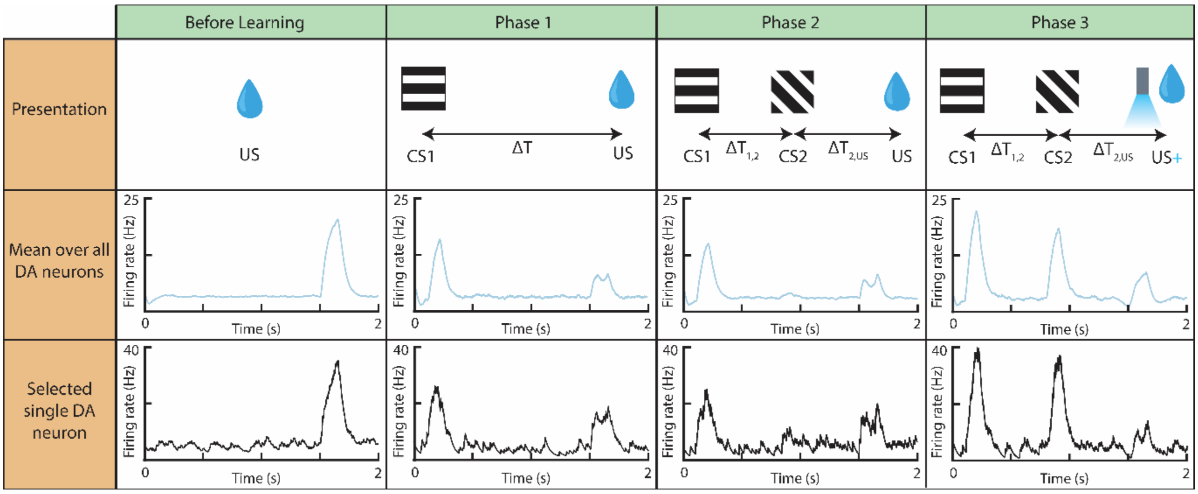

Supplemental Figure 8 – Expected Rewards Block Learning of New Cue-Reward Associations Unless Reward Magnitude is Increased

Each column marks a different phase of conditioning in a blocking/unblocking paradigm. Results shown are averaged over 25 trials. Top row, visual representation of protocol in given column. Middle row, mean over all DA neurons and trials for given presentation protocol. Bottom row, a selected single unit response averaged over trials of the given presentation protocol. Before learning, unpredicted rewards trigger dopamine release at t_{US}. In phase 1, CS1→US is learned, and DA neurons develop a response to CS1 and have suppressed their response to the US. In phase 2, CS1→CS2→US is presented, but a dopamine response fails to develop to CS2, as the US is already fully predicted by CS1 (and therefore CS2 is “blocked”). Phase 3 results in “unblocking” via increasing dopamine release at t_{US} – doing so recovers the CS2-evoked response and results in both stimuli becoming reward-predictive. Definitions: first conditioned stimulus (CS1), second conditioned stimulus (CS2), unconditioned stimulus (US).

Appendix

A Mathematical derivation of change of temporal integral of RPE over learning

Mathematical proof that the temporal integral of the reward prediction error is non-increasing over learning iterations of TD(0) learning. This proof is for the tabular form of TD, that is we assume a table of values. The notation $V_n(t)$ is the value for (discrete) time bin t, over the $n'th$ iteration of TD(0) learning. The time bins range from the initial staate $t = 1$ to the terminal state $t = T$. The reward prediction error of TD(0) has the form:

$$\delta_n(t) = r(t) + \gamma V_{n-1}(t+1) - V_{n-1}(t). \tag{A-1}$$

Here $r(t)$ is the reward such that the integral of sum over t is $\sum_t r(t) = \rho$, and $\gamma \leq 1$ is the discount factor. Using this δ_n, V_n is updated by:

$$V_n(t) = V_{n-1}(t) + \alpha\delta_n(t) \tag{A-2}$$

where α is the learning rate. We assume here a batch form so the update from V_{n-1} to V_n is done all at once after the terminal state. Lets now define the temporal sums over all time points from the initial point $t = 1$ to the terminal point $t = T$ for the error δ_n as $I\delta_n$ and for the value V_n as IV_n. Therefore:

$$IV_n = IV_{n-1} + \alpha I\delta_n \tag{A-3}$$

and

$$\begin{aligned} I\delta_n &= \rho + (\gamma-1)IV_{n-1} - \gamma V_{n-1}(1) = \rho + (\gamma-1)(IV_{n-2} + \alpha I\delta_{n-1}) - \gamma V_{n-1}(1) \\ &= \underbrace{(\rho + (\gamma-1)IV_{n-2})}_{I\delta_{n-1}+\gamma V_{n-2}(1)} + \alpha(\gamma-1)I\delta_{n-1} - \gamma V_{n-1}(1) \end{aligned} \tag{A-4}$$

therefore:

$$I\delta_n = I\delta_{n-1}\underbrace{(1-\alpha(1-\gamma))}_{\leq 1} - \gamma\left(V_{n-1}(1) - V_{n-2}(1)\right) \tag{A-5}$$

Note that if the initial condition $V_0(t)$ is such that it is smaller than the final value, and is monotonically increasing with t then the value function increases over iterations until the fixed point is reached from below, and therefore: $V_{n-1}(1) \geq V_{n-2}(1)$.
